# Alpha-1 Antitrypsin Inhibits Tumorigenesis and Progression of Colitis-Associated Colon Cancer through Suppression of Inflammatory Neutrophil-Activated Serine Proteases and IGFBP-3 Proteolysis

**DOI:** 10.3390/ijms232213737

**Published:** 2022-11-08

**Authors:** Qing Cai, Minsun Kim, Aki Harada, Michael O. Idowu, Gobalakrishnan Sundaresan, Jamal Zweit, Youngman Oh

**Affiliations:** 1 Department of Pathology, Virginia Commonwealth University, Richmond, VA 23298, USA; 2 Massey Cancer Center, Virginia Commonwealth University, Richmond, VA 23298, USA; 3 Department of Pediatrics, Jeonbuk National University Medical School, Jeonju 54907, Korea; 4 Department of Radiology, Virginia Commonwealth University, Richmond, VA 23298, USA; 5 Department of Biochemistry and Molecular Biology, Virginia Commonwealth University, Richmond, VA 23298, USA

**Keywords:** colitis-associated colon cancer, AAT, neutrophil, NSPs, IGFBP-3, IGFBP-3R, antitumor, anti-inflammation

## Abstract

Colitis-associated colon cancer (CAC) accompanies the massive infiltration of neutrophils during tumorigenesis and progression of CAC. Depletion of neutrophils in circulation results in significant inhibition of tumor incidence in CAC. However, the underlying mechanisms are largely unclear. In this study, we provide evidence for the crucial involvement of inflammatory neutrophil-activated serine proteases (NSPs) on the dysregulation of the anti-inflammatory and antitumor IGFBP-3/IGFBP-3R signaling axis in CAC using a chronic AOM/DSS mouse model. We also provide preclinical evidence for α1-antitrypsin (AAT) as a preventive and as a therapeutic for CAC. AAT administration not only prevented colitis-associated tumorigenesis but also inhibited established CAC. AOM/DSS treatment results in the significant activation of NSPs, leading to CAC through increased pro-inflammatory cytokines and decreased anti-inflammatory and antitumor IGFBP-3. Collectively, these data suggest that the NSPs proteolyze IGFBP-3, whereas AAT inhibits chronic colonic inflammation-induced NSP activity and subsequently suppresses IGFBP-3 proteolysis. Therefore, the anti-inflammatory and antitumor functions of the IGFBP-3/IGFBP-3R axis are restored. AAT mimicking small peptides also showed their inhibitory effects on NSP-induced IGFBP-3 proteolysis. These results suggest that targeting the NSP-IGFBP-3/IGFBP-3R axis using NSP inhibitors such as AAT and the AAT mimics and IGFBP-3R agonists could lead to novel approaches for the prevention and treatment of CAC.

## 1. Introduction

Colorectal cancer is the second most common cause of cancer deaths in the United States and the third most diagnosed cancer worldwide [1]. The initiation and progression of colorectal cancer is driven by a variety of factors including genetic mutations, epigenetic abnormalities and colonic inflammation such as inflammatory bowel disease (IBD)-Crohn’s disease and ulcerative colitis [2,3,4,5]. Recent studies further demonstrated that an elevated inflammatory neutrophil-to-lymphocyte ratio predicts a significantly higher risk of death in colorectal cancer [6,7]. CAC is the subtype of colorectal cancer that is associated with IBD and the cumulative risk of CAC can be as high as 40% after 40 years of chronic IBD [8,9]. Recent studies demonstrated that a massive infiltration of neutrophils occurs in the lamina propria and submucosa during the progression of CAC [10], while depletion of neutrophils in circulation results in a significant reduction of the number and size of the tumors [10]. Furthermore, it has been shown that inflammatory neutrophil-induced serine proteinases (NSPs) such as neutrophil elastase (NE), cathepsin G (CG) and proteinase-3 (PR3) are not necessarily crucial for their antimicrobial activity, but involved in the pathogenesis of neutrophil-dependent inflammation and progression of chronic inflammatory diseases, particularly the development of IBD and further progression of CAC [10,11,12,13].

α1-antitrypsin (AAT), a member of the serine protease inhibitor (serpin) superfamily, is mainly produced by the liver and also by inflammatory cells, intestinal paneth cells as well as other types of epithelial cells [14]. The clinical significance of AAT is highlighted by a rare genetic disorder, AAT deficiency [14,15]. AAT deficiency manifests as several neutrophilic diseases associated with emphysema, liver cirrhosis, panniculitis, and systemic vasculitis. Recent studies have shown that AAT is a specific NSP inhibitor and exerts anti-inflammatory and tissue-protective effects [16]. It is further shown that AAT treatment attenuates colitis and chronic ileitis through the suppression of cytokine production and enhanced intestinal barrier function, thus strongly suggesting therapeutic potential of AAT treatment for IBD [17]. Furthermore, several studies have shown significant changes of AAT in serum and tumor tissue in a variety of cancers including colorectal cancer [18,19,20,21].

Insulin-like growth factor binding protein-3 (IGFBP-3), a major IGFBP species in circulation, has been demonstrated to have not only direct antitumor functions in human cancers but also anti-inflammatory properties in normal cells through the activation of a specific receptor, IGFBP-3R [22,23,24,25,26]. Recent large prospective studies showed a strong correlation between low serum IGFBP-3 levels and increased colorectal cancer risk [27,28,29]. Further studies have shown a significant reduction of IGFBP-3 protein in IBD [30,31] and the antitumor effects of IGFBP-3 in CAC using IGFBP-3 transgenic mice [32]. Interestingly, it was shown that neutrophil serine proteinases NE, CG and PR3 are IGFBP-3 specific proteases and further shown that there is a positive correlation among IGFBP-3 proteolysis, neutrophil serine proteinase activity, and chronic inflammatory manifestation such as insulin resistance, BMI, TNF-α and IL-8 [33,34,35]. 

In the present study, we first assessed the preventive and therapeutic potential of AAT for CAC using a chronic dextran sulfate sodium (DSS) colitis-azoxymethane (AOM) mouse model. We next characterized the anti-tumor IGFBP-3/IGFBP-3R signaling axis in colorectal cancer cells. Finally, we demonstrated the effect of NSPs and NSP inhibitors on the anti-inflammatory and anti-proliferative actions of the IGFBP-3/IGFBP-3R axis in CAC. 

## 2. Results

### 2.1. AAT Prevents Colitis-Associated Tumorigenesis

**Figure 1:**
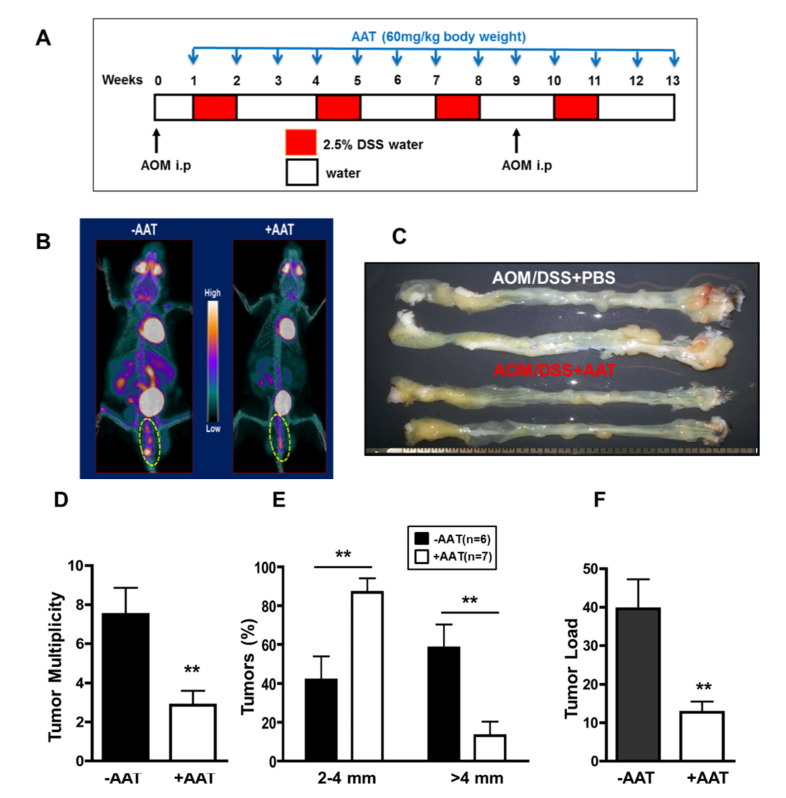

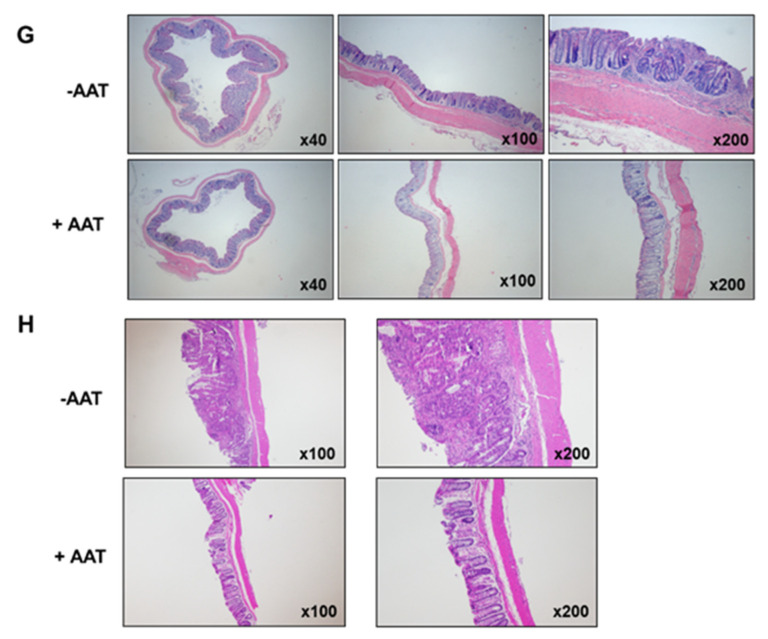
Preventive effects of AAT on CAC development. (**A**) Schematic overview of preventive protocol for AAT administration in AOM/DSS-treated mouse. Mice were injected with AOM (day 2 and day 65) followed by four cycles of 2.5% DSS in drinking water. ^18^F-FDG PET/CT image and colon tumors were analyzed on day 91. (**B**) A representative 3D Maximum Intensity Projections of AOM/4cycle DSS (total 91 days) treated mice with/without AAT administration. Four regions within the yellow circle show high FDG uptake and are prominent in the colon of no AAT administration (**left**) while showing only one region with low FDG uptake in AAT administrated colon (**right**) in AOM/DSS-treated mouse. Gamma counting of the entire colon was 8.8% ID/g (**left**) and 5.6 %ID/g (**right**). FDG %ID/g = FDG uptake percent injected dose per gram of tissue. (**C**) Colons were removed and examined for macroscopic changes. Tumors were confined to the middle and distal colon. Tumor multiplicity (**D**), tumor distribution (**E**), and average tumor load (**F**) were determined. Results, mean ± SD [n = 6 (−AAT), n = 7 (+AAT); **, *p* < 0.001]. (**G**,**H**) Anti-inflammatory and antitumor effects of AAT during initiation and progression of CAC were analyzed after treatment of AOM/2cycle DSS (total 47 days) (**G**) as well as AOM/4cycle DSS (total 91 days) (**H**). Colon tissue was further fixed and stained with hematoxylin/eosin. top, Control colon tissue without AAT treatment. Bottom, AAT treated colon tissue. In PBS-treated control colon tissue, the mucosa is expanded by glandular hyperplasia, mixed inflammation, and TIS formation. The submucosa is also expanded by inflammation. Original magnification, ×4, ×10 and ×20.

Neutrophils have been implicated in the initiation and progression of CAC [10,12]. AAT is known to exert anti-inflammatory and tissue-protective effects, at least (if not only) through inhibition of neutrophil-activated serine proteases (NSPs) such as neutrophil elastase (NE), cathepsin G (CG) and proteinase-3 (PR3) [36]. To examine the involvement of NSPs in CAC development and the therapeutic potential of AAT for CAC, we began to assess the preventive effect of AAT administration in AOM-DSS murine preclinical CAC models. As shown in Figure 1A, 12.5 mg/kg AOM was injected intraperitoneally on day 2 and, after a 5-day recovery period, the mice were started on the first of 4 cycles of 2.5% DSS ad libitum. Each cycle was 7 days in length and was separated by a 14-day recovery period. A second injection of AOM was performed on day 65 before the last cycle. Aralast, the clinical formulation of AAT (60 mg/kg body weight), was administrated intraperitoneally at the beginning of DSS treatment and weekly injection was continued until day 84. After 7 days of recovery, PET/CT image analysis was performed, and the mice were sacrificed afterwards. Representative ^18^F-FDG PET/CT images clearly show four regions with high ^18^F-FDG accumulation in the PBS administrated colon whereas only one region had low ^18^F-FDG accumulation in the AAT administrated colon in the AOM-DSS treated mice (Figure 1B). The ^18^F-FDG % ID/g values were 8.8% and 5.6%, respectively. Macroscopic examination demonstrates that tumors were confined to the middle and distal colon and tumor nodules in the colon of the mice administrated with AAT were significantly decreased (Figure 1C). AAT administration results in a significant reduction in tumor number (Figure 1D, *p* < 0.01), tumor size (Figure 1E, *p* < 0.01) and tumor load (Figure 1F, *p* < 0.01). Furthermore, microscopic analysis using hematoxylin/eosin stained tissue clearly demonstrates that AAT administration significantly reduced colonic inflammation, mucosa expansion, inflammatory cell infiltration, muscle thickening and intramucosal adenocarcinoma (TIS) formation compared with PBS-treated controls in early stage (total 47 days, Figure 1G) as well as at a later stage (total 91 days, Figure 1H). Thus, AAT administration decreased histological evidence of tissue damage and of tumor growth, and decreased inflammatory neutrophil/macrophage infiltration during CAC progression.

### 2.2. AAT Inhibits Established CAC

**Figure 2:**
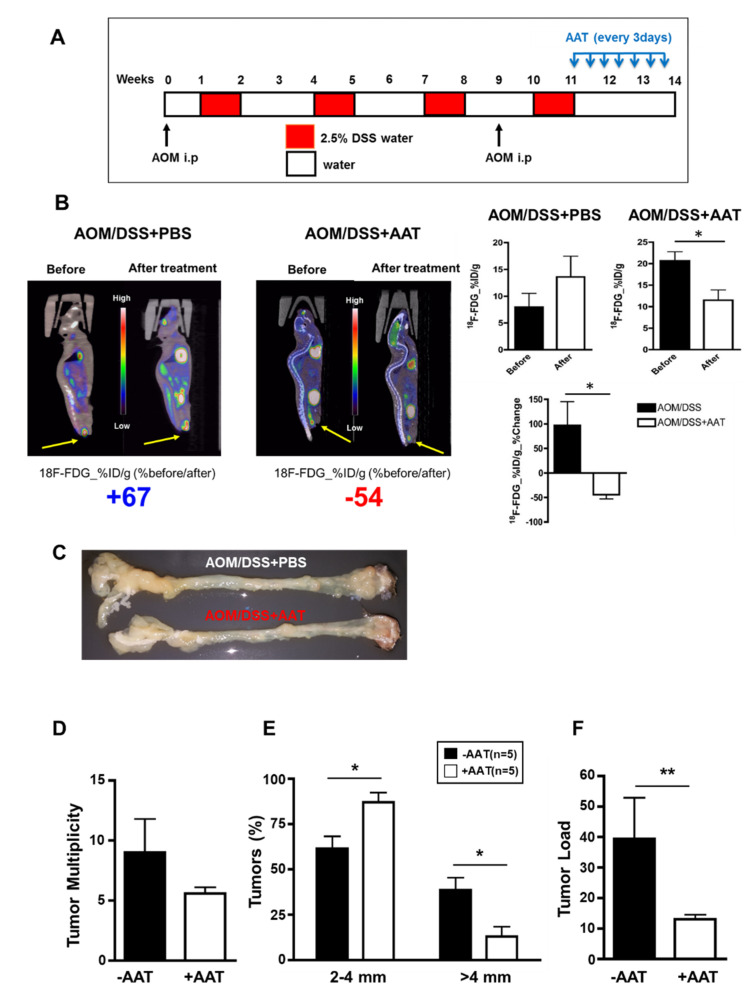

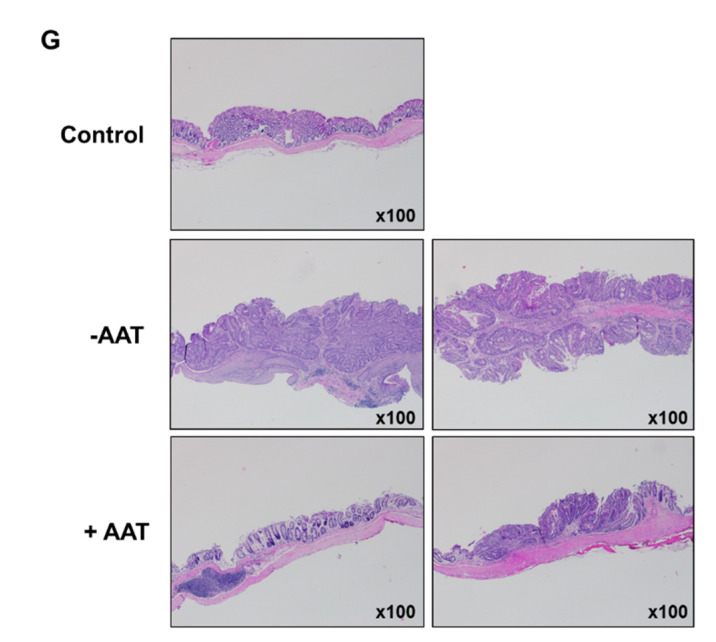
Therapeutic effects of AAT on established CAC. (**A**) Schematic overview of end-stage disease protocol for AAT administration in AOM/DSS-treated mice. Mice were injected with AOM (day 2 and day 65) followed by four cycles of 2.5% DSS in drinking water. AAT was administrated every 3 days after the last DSS cycle (day 77) for 18 days and tumors were examined on day 98. (**B**) **left**, A representative 18F-FDG PET/CT image of AOM/DSS-treated mice before (day 75) and after (day 98) AAT administration. **Right**, Gamma counting of entire colon was analyzed before and after treatment. The % change of FDG %ID/g of the before and after results between AOM/DSS-treated mice and AOM/DSS-treated mice with AAT administration was further analyzed. Results, mean ± SD [n = 3 (−AAT), n = 5 (+AAT); *, *p* < 0.05. (**C**) Macroscopic changes in colon by AAT administration: Tumor multiplicity (**D**), tumor distribution (**E**), and average tumor load (**F**) were determined. Results, mean ± SD [n = 5 (−AAT), n = 5 (+AAT); *, *p* < 0.05; **, *p* < 0.01]. (**G**) Colon tissue was fixed and stained with hematoxylin/eosin on day 98. Top, control colon tissue. AOM/DSS-treated colon tissue without (middle) and with (bottom) AAT administration. AOM/DSS and AAT-treated mice show reduced glandular hyperplasia, mixed inflammation and intramucosal adenocarcinoma formation, which is comparable to no AOM/DSS-treated control mice. Original magnification, ×10.

**Figure 3:**
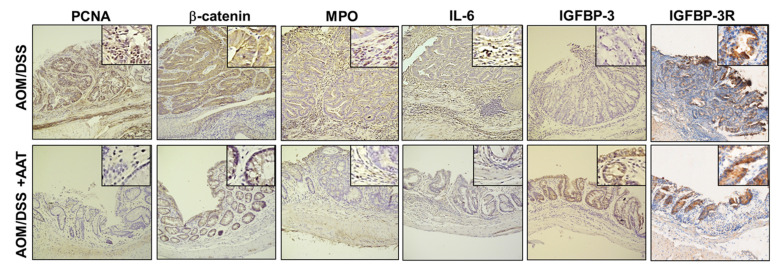
Effects of AAT on cytokine and antitumor factor production in CAC. Immunohistochemical staining for PCNA, β-catenin, MPO, IL-6, IGFBP-3 IGFBP-3R identified in colons from AOM/DSS-treated mice versus AOM/DSS and AAT-treated mice using an end-stage disease protocol as in Figure 2. Original magnification, ×20.

To investigate therapeutic potential of AAT in established CAC, AAT was administrated in AOM-DSS treated mice using an end-stage disease protocol (Figure 2A). AAT was treated every 3 days after the last DSS cycle (day 77) for 18 days. PET/CT image analysis was performed on day 75 before the first AAT administration and day 98 after completion of AAT treatments (Figure 2B). In the control-treated mice, ^18^F-FDG uptake significantly increased from 7.97 ± 2.56 %ID/g on day 77 to 13.59 ± 3.86 %ID/g on day 98 whereas in the AAT-treated mice, ^18^F-FDG uptake decreased from 20.62 ± 2.12 %ID/g on day 77 to 11.51 ± 2.37 %ID/g on day 98. When comparing between control-treated mice and AAT-treated mice, %ID/g values increased by 90% on day 98 compared with baseline while %ID/g values decreased by 50% on day 98 compared with baseline (*p* < 0.05) in the AAT-treated mice. Macroscopic examination indicated an antitumor effect of AAT showing a significant decrease of tumor nodules in the colon (Figure 2C). Similar to the results from AAT administration with a preventive protocol, AAT administration results in a significant reduction in tumor number (Figure 2D), tumor size (Figure 2E, *p* < 0.05) and tumor load (Figure 2F, *p* < 0.01) in mice harboring established CAC. Microscopic analysis demonstrates that AAT administration significantly reduced glandular hyperplasia, mixed inflammation and TIS formation compared with PBS-treated AOM/DSS mice (Figure 2G). Further IHC data demonstrates significant suppression of TIS formation as well as proliferating cell nuclear antigen (PCNA), β-catenin, myeloperoxidase (MPO) and IL-6 in colon tissue (Figure 3). In addition, AAT treatment results in a significant increase of IGFBP-3 in colon tissue whereas IGFBP-3R levels show no significant changes with or without AAT administration. These findings clearly demonstrate that AAT not only inhibits DSS-mediated colonic inflammation, but also suppresses AOM-DSS-established colon tumor growth in part, if not only, through the increase of antitumor and anti-inflammatory IGFBP-3 levels in colon tissue.

### 2.3. Treatment Decreases Circulating NSPs and IGFBP-3 Proteolysis in Established CAC

**Figure 4:**
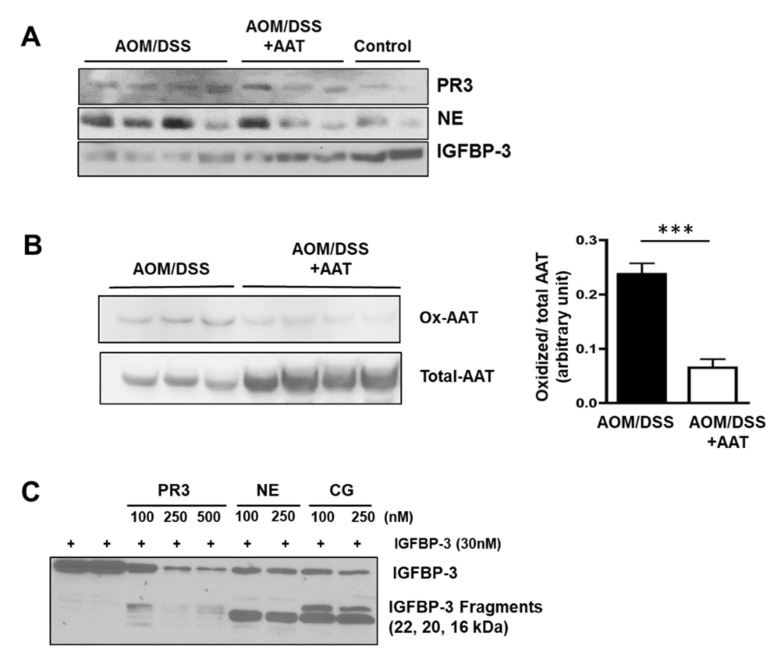

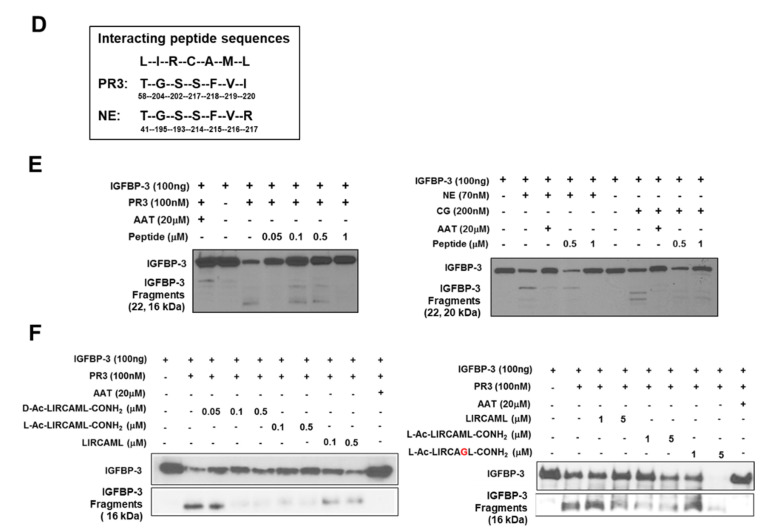
AAT suppresses increased serum neutrophil serine proteases PR3 and NE and subsequent IGFBP-3 proteolysis in AOM/DSS mice. (**A**) Representive WIB of sera from 4 AOM/DSS treated mice, 3 AOM/DSS+AAT treated mice and 2 control mice. AOM/DSS treated mice show increased PR3, NE and IGFBP-3 proteolysis in circulation whereas AAT administration results in significant inhibition of serum PR3, NE and increase of intact IGFBP-3 in circulation. (**B**) AAT administrated AOM/DSS mice show decreased level of oxidized-AAT but increased total AAT in circulation. ***, *p* < 0.001. (**C**) NSPs proteolyze IGFBP-3. Recombinant non-glycosylated human IGFBP-3 protein (30 nM) was proteolyzed by treatment with 100, 250 and 500 nM of PR3, 100 and 250 nM NE or CG for 20 min. at 37 °C. (**D**–**F**): AAT and AAT mimic inhibit NSP-induced IGFBP-3 proteolysis. Based upon the sequence identity among NSPs at a putative binding site of AAT and elafin, a 7 amino acid-long peptide (LIRCAML) was generated (**D**). IGFBP-3 proteolysis by PR-3 (**left** figure) and NE, CG (**right** figure) was inhibited by AAT (20 μM) and different concentrations of an AAT mimic (0.05, 0.1, 0.5 and 1 μM) (**E**). Few IGFBP-3 proteolytic fragments were detected in 20 μM AAT treated or 1 μM AAT mimic treated samples. Anti-protease activity of L- and D-form of N- and C-terminal modified AAT mimics (**left** figure) (**F**). Nonfunctional mutant lost anti-protease activity (**right** figure).

Since AOM/DSS treatment results in a significant increase of neutrophil infiltration and neutrophil azurophilic granules MPO levels in colon tissue, we have further analyzed serum circulating NSPs, endogenous AAT and IGFBP-3 in AOM/DSS mice with/without AAT administration. As shown in Figure 4A, significantly increased levels of PR3 and NE in circulation were observed in AOM/DSS treated mice compared to those in control mice whereas both PR3 and NE were significantly decreased in AAT administrated AOM/DSS mice. Moreover, the AAT treated mice showed increased intact IGFBP-3 in circulation which was significantly decreased in AOM/DSS treated mice. Furthermore, AAT administrated AOM/DSS mice showed a significant decrease of nonfunctional oxidized AAT (Ox-AAT) but increased total AAT in circulation (Figure 4B).

The ratio of the oxidized over total AAT was decreased by up to 70% in AAT administrated AOM/DSS mice. Observed reduction of the oxidized form of AAT, which lacks serine protease inhibitory activity [37,38], indicates that AAT administrated AOM/DSS mice would have increased functional AAT in circulation, thereby more potently inhibiting NSP-induced IGFBP-3 proteolysis. In order to confirm that the decreased level of IGFBP-3 in circulation in AOM/DSS mice is attributed to increased NSP-induced proteolysis, an in vitro proteolysis assay was performed using recombinant non-glycosylated human IGFBP-3 protein. Recombinant IGFBP-3 (30 nM) was proteolyzed by treatment with PR3, NE and CG in a dose-dependent manner (Figure 4C). Intact 28 kDa IGFBP-3 was gradually reduced by increased concentrations of NSPs, and concomitantly 16, 20 and 22 kDa IGFBP-3 proteolytic fragments were readily detected. It is our great interest to generate and characterize the novel AAT mimicking peptide inhibitors of NSPs since current clinical NSP inhibitors including Aralast are the natural forms of AAT purified from human plasma. Based upon the sequence identity among NSPs on a putative binding site of AAT and elafin, a secretory leukocyte protease inhibitor [39], we have generated a series of small peptides and further identified a 7 amino acid-long AAT mimic peptide (RS Synthesis, KY) that blocks NSP binding to the substrates and their protease activity (Figure 4D). This small AAT mimic peptide (LIRCAML) shows potent inhibitory effects on PR-3-induced IGFBP-3 proteolysis at concentrations ranging from 0.05 μM to 1 μM in a dose-dependent manner (Figure 4E, *left panel*). Even treatment with the AAT mimic peptide at the concentration of 0.05 μM resulted in more than 50% inhibition of IGFBP-3 proteolysis induced by 100nM PR3 treatment (lane 4). The inhibitory effect of the AAT mimic on PR3 activity at the concentration of 1 μM was comparable to that of 20 μM AAT (lanes 1 and 7). This small peptide AAT mimic abrogates not only PR-3-induced proteolysis, but also NE- and CG-induced IGFBP-3 proteolysis (Figure 4E, *right panel*). The inhibitory effect of the AAT mimic on NE and CG activities at the concentration of 1 μM was also comparable to that of 20 μM AAT (lanes 3 vs. 5 and 8 vs. 10). We have further generated the N- and C-terminal modified peptide (Ac-LIRCAML-CONH_2_) and shown that these are potent NSP inhibitors and block NSP-induced IGFBP-3 proteolysis (Figure 4F, *left panel*). Both L- and D-form of Ac- LIRCAML-CONH_2_ show a similar protective effect on NSP-induced IGFBP-3 proteolysis, which was comparable to an unmodified AAT mimic peptide (LIRCAML). Furthermore, the AAT mimic with a methionine to glycine substitution showed no inhibitory effect on NSP protease activity and even enhanced NSP protease activity at the concentration of 5 μM (Figure 4F, *right panel*). Taken together, these findings suggest that AAT and the small peptide AAT mimics are potent NSP inhibitors and may block NSP-induced IGFBP-3 proteolysis, thereby restoring IGFBP-3′s antitumor and anti-inflammatory function in CAC.

### 2.4. Anti-Proliferative and Anti-Inflammatory IGFBP-3/IGFBP-3R Signaling Is Functional in Colon Cancer Cells

**Figure 5:**
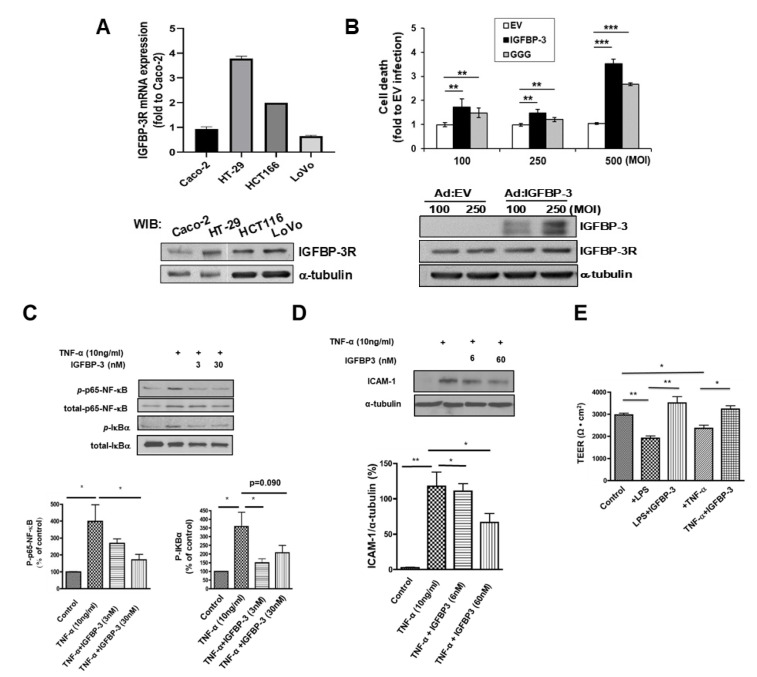
Anti-proliferative and anti-inflammatory effects of the IGFBP-3/IGFBP-3R axis in colon cancer cells. (**A**) Basal expression of IGFBP-3R at mRNA (**top**) and protein (**bottom**) levels analyzed by Quantitative RT-PCR and WIB, respectively. The mRNA data are presented as fold of change compared to Caco-2. The values of Caco-2 were considered as 1. The cell lysates were subjected to WIB for IGFBP-3R expression. The data represent mean ± SE, n = 3 in duplicate. Heterogeneous expression of IGFBP-3R was observed. (**B**) Data demonstrating IGFBP-3-induced apoptosis in HT-29 cells (**top**). Cell death assay was performed two days after the infection. Apoptotic cell death was measured using the Cell Death Detection ELISA. The value for Ad:EV at each MOI was considered as 1. The data represent mean ± SE, n = 3 in duplicate. *, *p* < 0.05; **, *p* < 0.01; ***, *p* < 0.001. Representative immunoblot analysis for IGFBP-3 and IGFBP-3R after infection of Ad:IGFBP-3 (**Bottom**). Ad:EV: adenoviral plasmids with empty vector; Ad:IGFBP-3: with IGFBP-3 cDNA; Ad:IGFBP-3^GGG^: with IGFBP-3^GGG^ mutant cDNA. Inhibitory effects of IGFBP-3 on TNF-α-induced NF-κB activity (**C**) and subsequent induction of ICAM-1 expression (**D**) in HT29 colon cells. n = 3. (**E**) Protective role of IGFBP-3 on LPS- or TNF-α-induced disruption of colonic epithelial barrier function. Measurement of TEER across confluent intestinal monolayers treated with LPS (1mM) or to TNF-α (100 ng/mL) for 2 days in the presence/absence of IGFBP-3 (30 nM) using a Millicell-ERS voltohmmeter. n = 3 in duplicate.

We have further investigated the existence of the IGFBP-3/IGFBP-3R system in CRC. IGFBP-3R expression was detected at mRNA and protein level in most colon cancer cells (Figure 5A). When IGFBP-3 or IGFBP-3^GGG^ (an IGFBP-3 mutant which only binds to IGFBP-3R but not IGFs [25]) was overexpressed, a significant induction of apoptosis was observed (Figure 5B), suggesting the existence of a functional IGFBP-3/IGFBP-3R system in CRC. As shown in Figure 5C, treatment with recombinant IGFBP-3 inhibits TNF-α-induced NF-κB activity in HT29 colon cells. While 10 ng/mL TNF-α treatment resulted in increase of phosphorylation of p65 NF-κB (400%, *p* < 0.05) and IKB-α (350%, *p* < 0.05), IGFBP-3 significantly inhibited TNF-α-induced phosphorylation of both proteins (*p* < 0.05). IGFBP-3 further suppressed NF-κB regulated ICAM-1 expression induced by TNF-α treatment (Figure 5D, *p* < 0.05). Moreover, 30 nM IGFBP-3 treatment prevented the LPS- and TNF-α-induced transepithelial electric resistance (TEER) loss (*p* < 0.01, *p* < 0.05, respectively), suggesting that IGFBP-3 prevents intestinal barrier dysfunction, which is a pivotal characteristics of IBD [40] (Figure 5E). These results strongly suggest that the IGFBP-3/IGFBP-3R axis is an important antitumor and anti-inflammatory signaling pathway and is impaired in CRC.

## 3. Discussion

It is well known that chronic inflammation contributes to tumor initiation, progression, invasion and metastasis in a variety of tumors [41,42]. CAC is the subtype of colorectal cancer that is associated with IBD and the cumulative risk of CAC can be as high as 40% after 40 years of chronic IBD [8,9]. Recent studies have demonstrated that massive infiltration of neutrophils occurs in the lamina propria and submucosa during the progression of CAC, with depletion of neutrophils in circulation resulting in a significant reduction of the number and size of the tumors [10], suggesting that colonic inflammation-activated neutrophils play an indispensable role in the initiation and progression of CAC [10,12]. Moreover, NSPs produced by activated neutrophils play a critical role for neutrophil-dependent inflammation and progression of chronic inflammatory diseases, particularly the development of IBD and the further progression of CAC [10,11,12,13].

On the other hand, AAT is a specific NSP inhibitor and exerts anti-inflammatory and tissue-protective effects [15]. AAT treatment also attenuates colitis and chronic ileitis through suppression of cytokine production and enhanced intestinal barrier function, thus strongly suggesting its therapeutic potential in IBD [17]. Although AAT is the most prevalent human anti-protease, it is sensitive to oxidation. Recent studies demonstrated that oxidized AAT (Ox-AAT) significantly loses the ability to protect the lungs from neutrophil elastase [37,38]. Our results further demonstrate an increased level of Ox-AAT, accompanying significantly increased levels of PR3 and NE in circulation in AOM/DSS treated mice, indicating that oxidative stress induced by chronic colonic inflammation could reduce NSP inhibitor activity of AAT during the progression of CAC.

IGFBP-3 has not only direct antitumor functions in human cancers, but also anti-inflammatory properties in normal cells through the activation of a specific receptor, IGFBP-3R [22,23,24,25,43]. Bioinformatics analysis of TCGA data shows IGFBP-3 mRNA levels are significantly lower in breast, liver and cervical cancers, while slightly increased in colorectal cancer (CRC) compared to normal tissue. Further studies have shown the lack of association of IGFBP-3 gene polymorphisms with CRC [44]. However, recent large prospective studies show a strong correlation between low serum IGFBP-3 level and increased CRC risk [27,28,29]. In addition, a significant reduction of IGFBP-3 protein has been observed in IBD [30,31]. Taken together with the current study demonstrating decreased functional IGFBP-3 in circulation due to NSP-induced IGFBP-3 proteolysis in a CAC mouse model, these findings suggest that systemic IGFBP-3 appears to be critical for the pathogenesis/pathophysiology of both colonic inflammation and CRC. Indeed, recent studies demonstrated the antitumor effects of IGFBP-3 in CAC using IGFBP-3 transgenic mice [32]. However, the underlying mechanisms of regulation of the IGFBP-3/IGFBP-3R axis and both its anti-inflammatory and antitumor action in CRC are largely unknown.

**Figure 6:**
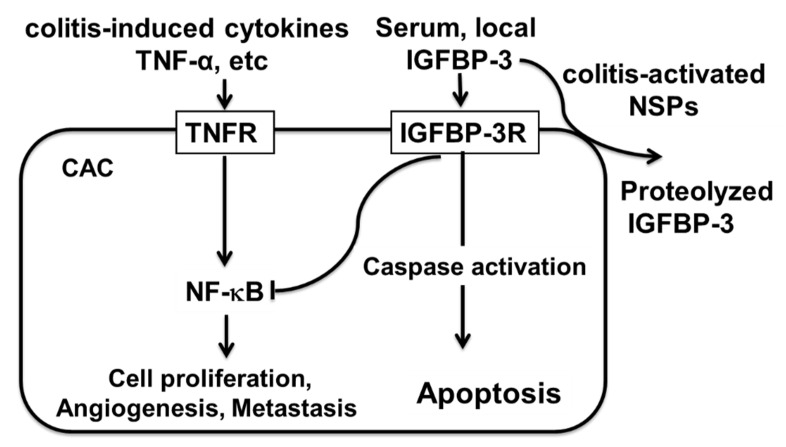
Central hypothesis: Colonic inflammation-induced NSPs proteolyze IGFBP-3 in circulation, thereby inhibiting endocrine/paracrine/autocrine-derived antitumor and anti-inflammatory actions of IGFBP-3 in CAC. IGFBP-3 activates IGFBP-3R and induces caspase-dependent apoptosis as well as inhibiting colitis-induced NF-κB-signaling, thereby resulting in suppression of CAC progression.

Although CAC development is an extremely complex process, it is well accepted that TNF-α-activated NF-κB signaling and IL-6-activated STAT3 signaling pathways take center stage for the development of CAC [11,45,46,47,48]. In particular, NF-κB is recently considered as a potential molecular bridge between tumor cells and inflammatory cells [49]. Since our previous studies demonstrated that the IGFBP-3/IGFBP-3R system suppresses tumor- and TNF-α-induced NF-κB activation and NF-κB-regulated genes including ICAM-1, VCAM-1, MCP-1 and IL-6 in a variety of cancers and inflammatory diseases [22,23,24,25,43,50], this indicates that colitis-induced activation neutrophils and subsequent activation of NSPs may lead to CAC through the increased production/activity of proinflammatory cytokines and decreased level of intact biologically-active IGFBP-3 in circulation and in colon tissue. However, treatment of NSP inhibitors such as AAT and the small peptide inhibitors may block NSP-induced IGFBP-3 proteolysis, thereby restoring the anti-inflammatory and antitumor IGFBP-3/IGFBP-3R system and further ameliorating neutrophil-activated cytokine functions such as activation of the IL-1β/IL-6 axis in CAC [12]. Thus, current findings further suggest therapeutic potential of AAT and small peptide inhibitors as well as IGFBP-3R agonists such as protease-resistant IGFBP-3 and IGFBP-3R agonist antibodies for CAC (Figure 6).

In summary, this study provides in vitro and in vivo experimental evidence for the pathophysiological significance of the NPS-IGFBP-3/IGFBP-3R axis in CAC and Aralast (a clinical formulation of AAT), the novel small peptide inhibitor (Ac-LIRCAML-CONH2), and IGFBP-3R agonists as preventive and therapeutic interventions for CAC.

## 4. Materials and Methods

### 4.1. Ethical Statement

Animal study protocols were reviewed and approved by the Institutional Animal Care and Use Committee at Virginia Commonwealth University (IACUC # AD10000931). 

### 4.2. Mice

C57BL/6 mice were purchased from the Jackson Laboratory (Bar Harbor, ME, USA), and were fed with chow diet (Lab Diet # 5001, calories provide by fat 13.5%, protein 28.5%, carbohydrates 58%, LabDiet, St. Louis, MO, USA). For a mouse model of colitis-associated cancer, mice were injected intraperitoneally (ip) with AOM (12.5 mg/kg body weight; Millipore Sigma St. Louis, MO, USA) diluted in PBS (ThermoFisher Scientific, Waltham, MA, USA). After 5 days, 2.5% DSS (MP Biomedicals, Irvine, CA, USA) was given in the drinking water for 7 days, followed by 14 days of regular water. This 21-day-cycle was repeated three times, and at day 65, the AOM/DSS cycle was repeated. In order to assess AAT as preventive intervention for CAC, AOM-DSS-treated mice were administrated with clinical formulation of AAT, Aralast (60 mg/kg body weight, ip, weekly, Baxter, Deerfield, IL, USA) or PBS (the same volume of AAT, ip, weekly) at the beginning of DSS treatment, and injection was continued until day 84. In order to assess the therapeutic potential of AAT as an intervention for CAC, AOM-DSS-treated mice were further administrated with Aralast (60 mg/kg body weight, ip, every three days) or PBS (same volume of AAT, ip, every three days) at one day after AOM/DSS induction, and then mice were treated with AAT for 2 months. For the PET/CT image acquisition and analysis, mice were intravenously injected with FDG and scanned prone in an Inveon PET/CT system (Siemens Medical Solutions, Erlangen, Germany)). Right after completion of each cycle, PET images were analyzed using Image Research workplace (Siemens Medical Solutions, Erlangen, Germany). Tracer accumulation within three-dimensional regions of interest was calculated as percent injected dose per gram (% ID/g) of tissue. After the colitis-associated cancer protocol, mice were fasted for 5 h, and then colon and blood were collected for downstream analysis. Macroscopic examination was performed to analyze tumor numbers and tumor size. Further microscopic analyses were performed for colonic inflammation, mucosa expansion, inflammatory cell infiltration, muscle thickening and intramucosal adenocarcinoma (TIS) formation.

### 4.3. Cell Lines and Reagents

The human colorectal carcinoma cell lines HT-29, LoVo, HCT116 and Caco-2 were purchased from ATCC (Manassas, VA, USA), and grown in high glucose DMEM (HT-29, LoVo, HCT116) and RPMI-1640 medium (Caco-2) supplemented with 10% fetal bovine serum. Cells were regularly screened for Mycoplasma using a MycoAlert Mycoplasma Detection Kit (Lonza, Basel, Switzerland)).

Rabbit anti-PR3, rabbit anti- NE, rabbit anti-CG, rabbit anti-AAT, rabbit anti-PCNA, rabbit anti-β-catenin, rabbit anti-MPO, rabbit anti-IL-6, rabbit anti-ICAM-1, HRP-anti-mouse and HRP-Anti-rabbit antibodies were purchased from Cell Signaling Technology (Danvers, MA, USA). Rabbit anti-p65-NF-κB, rabbit anti-p-p65-NF-κB, rabbit anti-IκBα, rabbit anti-p-IκBα, mouse anti-IGFBP-3 antibodies and Azoxymethane (AOM) were purchased from Santa Cruz Biotechnology (Dallas, TX, USA). Mouse anti-α-tubulin, rabbit anti-IGFBP-3R (TMEM219) were purchased from Millipore Sigma (St. Louis, MO, USA). Mouse anti-ox-AAT was purchased from Ikagaku Co. (Kyoto, Japan). LPS, TNF-α and NE were purchased from Sigma-Aldrich (St. Louis, MO, USA). PR3 and α1-antitrypsin (AAT) were purchased from Athens Research & Technology (Athens, GA, USA). Dextran sodium sulfate (DSS) and CG were purchased from MP Biomedicals (Irvine, CA, USA). Human alpha1-proteinase inhibitor Aralast NP was purchased from Baxter Healthcare Corp (Deerfield, IL, USA). Recombinant human IGFBP-3 protein was purchased from Abcam (Cambrideg, United Kingdom). Adenoviral vectors that express IGFBP-3 (Ad:GFBP-3), IGFBP-3^GGG^ (Ad:IGFBP-3^GGG^) and control empty vector (Ad:EV) were generated using AdEasy system (Quantum Biotechnologies, Montreal, QC, Canada) as described previously [24].

### 4.4. Immunohistochemistry

Mouse proximal colon tissues were fixed in 10% buffered formalin for 24 h at room temperature and subsequently embedded in paraffin. Tissues were sectioned at 5-μm thickness and deparaffinized. Sections were incubated in sodium citrate buffer and cooked in a pressure cooker for 10 min for antigen retrieval. Sections were then blocked with 5% BSA in TBS for one hour, followed incubation with the primary antibody at 4 °C overnight. After washing with TBS, sections were treated with secondary antibodies for 30 min at 37 °C, and color development was performed using the Vectastain ABC kit (Vector Laboratories, Newark, CA, USA). Sections were then counterstained with hematoxylin, and dehydrated. Images were obtained using a Zeiss AxioLab upright microscope (Carl Zeiss AG, Oberkochen, Germany) and Zeiss AxioCam ICc5 (Carl Zeiss AG, Oberkochen, Germany), and Zen2 software (Carl Zeiss AG, Oberkochen, Germany).

### 4.5. Transepithelial Electrical Resistance (TEER)

Measurement of TEER across confluent intestinal monolayers was performed in HT-29 cells treated with LPS (1 mM) or TNF-α (100 ng/mL) for 2 days in the presence/absence of IGFBP-3 (30 nM) using a Millicell-ERS voltohmmeter (Millipore Sigma, St. Louis, MO, USA). The ohmic resistance of a blank (culture insert without cells) was measured in parallel. To obtain the sample resistance, the blank value was subtracted from the total resistance of the sample. The final unit area resistance (Ω*cm^2^) was calculated by multiplying the sample resistance by the effective area of the membrane. n = 3 in duplicate.

### 4.6. Western Immunoblot Analysis

Cells were harvested in NP40 cell lysis buffer (Invitrogen, Waltham, MA, USA), and Halt Protease Inhibitor Cocktail (Thermo Scientific, Waltham, MA, USA). Equal amounts of protein in lysates and in the conditioned media or 1 μL of serum samples mixed with loading buffer were run on 12.5% reducing SDS-PAGE and electrotransferred onto Hybond-ECL nitrocellulose (Amersham Biosciences, Amersham, UK). Membranes were blocked in 5% nonfat milk, Tris-buffered saline, 0.1% Tween 20 (TBS-T). Primary antibodies were diluted in blocking solution and incubated on the membranes for 2 h at room temperature. Membranes were washed in TBS-T then incubated with HRP-conjugated secondary antibody, diluted 1:3000 for 1 h at room temperature, washed, and detected using the enhanced chemiluminescence reagents (Amersham GE Healthcare, Chicago, IL, USA), and signals were visualized by OPTIMAX X-Ray film Processor (Protec Medical, Oberstenfeld, Germany).

### 4.7. RNA Preparation and Quantitative Real-Time Polymerase Chain Reaction (RT-PCR)

Total RNA was extracted by using TRIzol (Invitrogen Thermo Fisher Scientific, Waltham, MA, USA) following the manufacture’s protocol. cDNA for quantitative real-time PCR (RT-PCR) was generated from equal amounts of RNA using the High Capacity cDNA Reverse Transcription Kit (Applied Biosystems, Waltham, MA, USA). cDNAs were amplified by RT-PCR using SYBR Green Mix. PCR reactions were carried out in CFX96 Touch/CFX384 Touch RT-PCR detection system (Bio-Rad Laboratories, Hercules, CA, USA). GAPDH was used as a housekeeping gene for an internal control. The sequences of the forward and reverse primers were as follows: human IGFBP-3 fwd, 5′-CAGAGCACAGATACCCAGAACTTC-3′; rev, 5′-CACATTGAGGAACTTCAGGTGATT-3′, human IGFBP-3R fwd, 5′-AGACAGGAACAAGACCCGGACATT-3′; rev, 5′-ATAGGCAGGTTCCTGCAGTCCTTT-3′, human GAPDH fwd, 5′-CCAATAGGCGCTCACTGTTCT-3′, rev, 5′-GCGAACTCACCCGTTGACT-3′, mouse IGFBP-3 fwd, 5′-GGGAGTGTGGAAAGCCAGGT-3′; rev, 5′-TCCCGCTTAGACTCGGAGGA-3′, mouse IGFBP-3R fwd, 5′-GCCCAGTGATGGGTGAGGAG-3′; rev, 5′-AGGCCACAGAGCAGAACCAG-3′, mouse GAPDH fwd, 5′-GTGTTCCTACCCCCAATGTGT-3′, rev, 5′-ATTGTCATACCAGGAAATGAGCTT-3′. The cDNA amplification was performed as described previously [50]. 

### 4.8. Statistical Analysis

Student *t*-tests were used to determine statistical significance. All data were presented as means ± standard deviation. *p* value less than 0.05 was considered significant.

## Data Availability

Data are available from the corresponding author on reasonable request.
